# Didn’t Plan One but got One: *Unintended* and *sooner-than-intended* Parents in the East and the West of Europe

**DOI:** 10.1007/s10680-021-09584-2

**Published:** 2021-05-27

**Authors:** Zuzanna Brzozowska, Isabella Buber-Ennser, Bernhard Riederer

**Affiliations:** 1 Vienna Institute of Demography, Austrian Academy of Sciences, Wittgenstein Centre, Vienna, Austria; 2 Department for Sociology, University of Vienna, Vienna, Austria

**Keywords:** Fertility intentions in Europe, East and West Europe, Childbearing intendedness, Realisation of fertility intentions, Social pressure on having a child, Anticipated costs and benefits of having a child, Generations and Gender Survey (GGS)

## Abstract

The realisation rates of short-term childbearing intentions are known to be consistently lower in post-socialist countries than in the rest of Europe. However, the East–West differences in the outcomes of intentions to postpone or forego (further) childbearing have not been previously examined. We employ two panel waves of the Generations and Gender Survey in six countries (three from Eastern and three from Western Europe), and, based on the short- and long-term fertility intentions expressed by respondents at the first survey wave, we classify the births occurring between two waves as intended, sooner-than-intended, or unintended. We find that in our study population of non-teenage respondents who had the same partner at both survey waves and a child between the two survey waves, between around 10% (Western European countries) and 30% (Eastern European countries) experienced an *unintended* or a *sooner-than-intended* birth. The East–West divide is largely driven by the share of *unintended* parents which is clearly higher in the post-socialist countries. However, the geographical pattern fades away once we control for the anticipated costs of having a child. Our study gives insight into East–West differences in attitudes to childbearing and into how they affect reproductive behaviour. It also offers methodological improvements of cross-national panel surveys designed to examine childbearing intentions that would allow for a more accurate assessment of childbearing intendedness.

## Introduction

In European demographic research, panel surveys have been extensively used to analyse the realisation of short-term intentions to have a child. The application of psychological theories defining intentions as “desires constrained by reality” (Miller, 1994, p. 228) or as an indication of “how hard people are willing to try, how much of an effort they are planning to exert in order to perform the behaviour” (Ajzen, 1991, p. 181) has sparked a widespread interest in examining how well short-term fertility intentions predict childbearing. As a result, there is rich evidence on the factors affecting this relationship (Dommermuth et al., 2015; Morgan & Rackin, 2010; Mynarska & Brzozowska, 2019; Philipov, 2009; Régnier-Loilier & Vignoli, 2011; Schoen et al., 1999; Thomson, 1997). In particular, it has been shown that the link between reproductive intentions and behaviour is consistently weaker in the post-socialist countries than in the rest of Europe (Brzozowska & Mynarska, 2018; Kapitány & Spéder, 2012; Spéder & Kapitány, 2014). This finding refers only to positive fertility intentions, i.e. the intention to have a child, and we do not know whether it extends also to negative fertility intentions, i.e. intentions to postpone or forego (further) childbearing.

Studying the outcomes of negative reproductive intentions is directly connected with the concept of childbearing intendedness. It is a highly complex construct, the meaning and the correct measurement of which have been debated for decades (Joyce et al., 2000; Pohlman, 1968; Santelli et al., 2003; Trussell et al., 1999; Westoff, 1980; Westoff & Ryder, 1977; Yeatman & Sennott, 2015). Demographers typically assume that when a woman becomes pregnant after engaging in voluntary sexual intercourse, the pregnancy is based on a rather conscious decision (Casterline & El-Zeini, 2007) and can be classified as intended (coming at the right time or later than desired), mistimed (coming earlier than desired), or unwanted (occurring despite being undesired).^1^ Thus, in a prospective set-up of a panel study, respondents who declared that they did not intend to have a child at the first survey wave, but who reported having a child by the second wave, are classified as having experienced an unintended (unwanted) or a sooner-than-intended (mistimed) birth. However, not all of these births are unwanted or mistimed, as some occurred following a change in a respondent’s childbearing intentions from negative to positive that happened between the measurement points (Régnier-Loilier & Sebille, 2017). Such a change occurs particularly often in response to changes in an individual’s life circumstances, like having found a new partner (Bernardi et al., 2015; Hayford, 2009; Heiland et al., 2008; Iacovou & Tavares, 2011; Ní Bhrolcháin et al., 2010).

Unlike in the United States, very few panel surveys aimed at analysing childbearing intendedness have been conducted in Europe (Baschieri et al., 2017; Koenig et al., 2006; Williams et al., 1999; Yeatman & Sennott, 2015). The current study employs cross-national panel data from the Generations and Gender Survey (GGS) to examine female and male respondents who experienced an *unintended* or *sooner-than-intended*^2^ birth between two survey waves in three European post-socialist countries (East) and three European countries without the state-socialist experience (West). To our knowledge, it is the first analysis providing comparative evidence on the prevalence of *sooner-than-intended* and *unintended* non-teenage mothers and fathers, their sociodemographic characteristics, and the risk factors associated with *sooner-than-intended* or *unintended* parenthood. Unlike previous single-country studies (Kuhnt & Trappe, 2013, 2016; Spéder & Kapitány, 2009), we treat respondents classified as unintended and sooner-than-intended parents as two separate groups, following Williams et al. (1999). We give insight into East–West differences in attitudes to childbearing and into how they affect reproductive behaviour. Finally, we offer methodological improvements of cross-national panel surveys designed to examine childbearing intentions that would allow for a more accurate assessment of childbearing intendedness.

## Previous Research: Prospective and Retrospective Approaches

In high-income countries, it is estimated that between 12% (Sweden) and around 30% (USA) of pregnancies carried to term are unplanned; i.e. are either mistimed or unwanted (Finer & Henshaw, 2006; Finer & Zolna, 2016; Stern et al., 2016). Compared to respondents who report that their pregnancies have been planned, those who indicate they have experienced an unwanted or mistimed birth show some degree of negative selection. They are, on average, of lower socioeconomic status, and are more likely to be in their teens or early twenties, and to lack a coresidential partner (Finer & Zolna, 2016; Font-Ribera et al., 2008; Goossens et al., 2016; Kågesten et al., 2015; Lukasse et al., 2015; Wellings et al., 2013). Many unplanned pregnancies result from a contraceptive failure rather than from contraceptive nonuse (Baird et al., 2018): in the USA, around half of unplanned conceptions occur while using contraceptives (Finer & Henshaw, 2006); in France, the corresponding figure is estimated at the level of 65–70% (Kågesten et al., 2015; Moreau et al., 2007).

All of these estimates and analyses of unplanned pregnancies and births are based on retrospective reports of women and men on the intendedness of their most recent or current pregnancy. As the retrospective approach suffers from the risk of recall error and *ex post* rationalisation, it tends to underestimate the number of unwanted and mistimed pregnancies (Koenig et al., 2006; see also Régnier-Loilier (2017) for how the reported intendedness can change over time). The prospective approach is not affected by these potential distortions, as it first assesses fertility intentions and does not check whether these intentions are realised until a later point in time. However, this method is based on the unrealistic assumption that the respondents’ fertility intentions remain stable between consecutive measurements. It has been shown that childbearing plans may change between two measurement points in response to “situational changes [in respondents’ lives] that had not been anticipated [at the first interview] and that made childbearing more desirable in the time period between interviews” (Williams et al., 1999, p. 225), like finding a new partner. Furthermore, women at older reproductive ages are known to have less stable and less certain fertility intentions (Bhrolcháin & Beaujouan, 2011; Rackin & Morgan, 2018). Thus, some of the pregnancies classified by the prospective approach as unintended or sooner-than-intended are in fact intended: they occur following changes in the respondents’ childbearing intentions that are not captured by the measurement.

Despite the inconsistencies between the retrospective and the prospective approaches, their empirical overlap is very strong (Rackin & Morgan, 2018). The findings of single-country studies using the prospective method have largely confirmed those of analyses based on the retrospective approach (Kuhnt & Trappe, 2016; Spéder & Kapitány, 2009). In the next section, we describe the set-up of the current study, which is cross-national and prospective. We begin with the individual characteristics that have been identified as risk factors of *unintended* and *sooner-than-intended* births and discuss how they potentially affect the prevalence of *unintended* and *sooner-than-intended* parents in the East and West of Europe. Then, we put forward arguments for why in the East we may find more *unintended* and *sooner-than-intended* parents relative to *intended* parents than in the West specifically when applying the prospective approach, i.e. a method that largely depends on how fertility intentions (both positive and negative) are realised.

## Present Study: *unintended* and *sooner-than-intended* Parents in East and West

It is important to note that, as finding a (new) partner or separating from a partner is strongly related to changes in childbearing intentions (Bernardi et al., 2015; Hayford, 2009; Heiland et al., 2008; Iacovou & Tavares, 2011; Ní Bhrolcháin et al., 2010), we focus on respondents who at both survey waves had the same partner, either coresidential (married or cohabiting couples) or in a Living-Apart-Together (LAT) arrangement. Furthermore, similarly to the two previous prospective studies (Kuhnt & Trappe, 2016; Spéder & Kapitány, 2009), there are no teenagers in our sample as their fertility intentions tend to be very unstable (Hayford, 2009) and, in the European context, teenage parents are a very specific, highly selective group.

### Sociodemographic Characteristics and Contraceptive Use

Previous research suggests that in comparison with *intended* parents, those classified as unintended or sooner-than-intended parents are more likely to be men than women because women face more severe consequences and higher opportunity costs of parenthood (Kuhnt & Trappe, 2016; Régnier-Loilier & Sebille, 2017). Furthermore, *unintended* parents tend to be older and have more children than both *sooner-than-intended* and *intended* parents (D’Angelo et al., 2004; Dutta et al., 2015; Exavery et al., 2014). By contrast, the *sooner-than-intended* parents are more likely to be younger and have fewer children. The exact meaning of “more” and “fewer” children may differ between the East and West of Europe because of their distinct parity progression patterns. Specifically, in post-socialist countries the transition rates to first birth are substantially higher while those to second and third birth are lower than in most Western European countries (Brzozowska et al., 2017; Zeman et al., 2018). Correspondingly, fewer Eastern European parents of one and, in particular, two children express an intention to have an additional child compared to Western European countries (Brzozowska & Mynarska, 2018; Bühler & Philipov, 2005; Régnier-Loilier & Vignoli, 2011). For this reason, it is likely that the effect of parity on becoming an *unintended* or *sooner-than-intended* parent is stronger in the East than in the West.

Observing negative selection into the groups of *unintended or sooner-than-intended* parents–namely lower levels of education and of employment–would be in line with the pattern found in retrospective studies. It could potentially indicate that the births were indeed unwanted or mistimed but it could as well hint on socioeconomic differences in, for example, opportunity costs of having children or projection capability. However, unlike studies based on the retrospective reports, the prospective analyses for Germany and Hungary (Kuhnt & Trappe, 2013, 2016; Spéder & Kapitány, 2009) found no difference in the educational attainment between *intended, sooner-than-intended* and *unintended* parents. The evidence on the role of employment is mixed. The Hungarian data indicated that respondents who were not in employment had higher chances of experiencing a *sooner-than-intended* or *unintended* birth than those with a job. By contrast, the German study identified such an effect for women only, and found no effect for men.

The role of contraceptive use could give some hints on whether the *unintended* or *sooner-than-intended* births were indeed unwanted or mistimed. The lower contraceptive uptake in Eastern (and Southern) Europe could result in a higher prevalence of *unintended* and/or *sooner-than-intended* parents in the East (and South) of Europe than in other parts of the continent (Dereuddre et al., 2016; Spinelli et al., 2000; United Nations, 2015). On the other hand, however, the existing evidence does not suggest that Eastern (and Southern) Europeans are less successful in controlling their fertility: the fertility levels in these two regions are among the lowest on the continent (e.g. Zeman et al., 2018).

### Realisation of Positive and Negative Fertility Intentions

Previous studies showed that the realisation rates of the intention to have a child are lower in post-socialist European countries than in the rest of Europe (Brzozowska & Mynarska, 2018; Kapitány & Spéder, 2012; Riederer & Buber-Ennser, 2019; Spéder & Kapitány, 2014). Ceteris paribus, lower realisation rates lead to a lower number of *intended* parents. Consequently, the shares of *unintended* and *sooner-than-intended* parents among respondents who had a child between the two survey waves may be systematically higher in the East than in the West of Europe.

The lower realisation of positive childbearing intentions has been explained by “the enduring persistence of social anomie” in post-socialist countries (Spéder & Kapitány, 2014, p. 394). Social anomie, as defined by Merton (1968), describes societies in which the normative and cultural system does not correspond to the structural (economic and institutional) system. After the collapse of communism, post-socialist countries underwent a rapid economic, political, and social transformation, which outpaced cultural and normative changes. It has been argued that the mismatch between the two main factors determining childbearing intentions–i.e. norms and values on the one hand, and structural conditions and constraints on the other–makes fertility plans more uncertain and unstable than in countries without the post-socialist transition experience (Bernardi et al., 2015). For instance, a German qualitative study showed a certain reluctance to make plans for the future among women living in the former East Germany as compared to women from the former West Germany (Bernardi & Keim, 2017). Unfortunately, the “social anomie” explanation has not been empirically tested in a quantitative manner. The data analysed in our study do not allow us to operationalise social anomie, either, but we look into the role of (un)certainty of fertility intentions and we construct variables that give insight into cultural and social perception of having children: the perceived social pressure on having a (further) child and the expected costs and benefits of a (further) child.

#### (Un)Certainty of Childbearing Intentions

It has been demonstrated that the link between reproductive intentions and behaviour is stronger for certain intentions than for uncertain ones: respondents *definitely* intending a child within the next two or three years are more likely to have one within that time horizon than respondents *probably* intending a child (Brzozowska & Beaujouan, 2020; Brzozowska & Mynarska, 2018; Régnier-Loilier & Sebille, 2017; Régnier-Loilier & Vignoli, 2011). Because of a more fragile economic environment along with the experience of a turbulent socioeconomic transition and the resulting different life planning strategy (Bernardi & Keim, 2017) Eastern Europeans may more often be uncertain about their intentions than Western Europeans (see also evidence on rising uncertainty of fertility intentions in response to financial difficulties in Testa and Gietel-Basten (2014)). In addition, respondents unsure about their childbearing plans are more likely to change them between the survey waves (Jones, 2017). This holds for both positive and negative fertility intentions (Buber-Ennser, 2014).

#### Social Pressure on Having a (Further) Child

Social pressure is known to strongly and positively affect the intention to have a child (Balbo & Mills, 2011; Billari et al., 2009; Buber-Ennser & Fliegenschnee, 2013; Dommermuth et al., 2011). Its effect on the realisation of fertility intentions is less explored. According to the Theory of Planned Behaviour (TPB), social pressure influences childbearing solely through intentions (Ajzen & Klobas, 2013; Klobas & Ajzen, 2015). We do not negate this assumption but point out another way in which social pressure may affect the realisation rates: it may “inflate” the intention to have a child and in this way contribute to the lower realisation of fertility intentions. What makes this assumption credible is the fact that countries with lower realisation rates are those where the perceived pressure on having a child is typically higher: the familistic societies of post-socialist (and Southern) Europe (Balbo & Mills, 2011; Calzada & Brooks, 2013; Mair, 2013).

#### Expected Costs and Benefits of Having a (Further) Child

Prior studies repeatedly showed that the expected costs and benefits of having a (further) child are important predictors of childbearing intentions: individuals who intend to have a child typically report higher benefits and lower costs than those with no childbearing intention (Billari et al., 2009; Buber-Ennser & Fliegenschnee, 2013; Dommermuth et al., 2011). By contrast, the evidence on how they affect the outcomes of reproductive plans is scant. Findings for Norway suggest that the expected costs and benefits do not affect the realisation of positive childbearing intentions (Dommermuth et al., 2015). However, in a cross-national context they may actually play a non-negligible role: depending on the economic, institutional, and sociocultural context, respondents with the same fertility intentions may assess the costs and benefits differently, which may translate into disparities in the outcomes of their fertility intentions. For instance, Eastern Europeans may evaluate the benefits of having a child similarly to Western Europeans but they are likely to be more pessimistic about the costs. Compared to the West, families in the East face tougher conditions: part-time jobs are extremely rare, the economic resources (e.g. salaries and housing) are more modest, and the cushion offered by the welfare state is rather thin. All these factors are likely to increase the anticipated costs of a (further) child which may lower the realisation of positive fertility intentions and, consequently, the number of intended parents. On the other hand, they may also prevent individuals from admitting they would like to have a (further) child in the near future or make their intentions more uncertain.

### Hypotheses

The shares of *sooner-than-intended* and *unintended* parents are higher in the East than in the West (H1). We hypothesise that while contraceptive use and sociodemographic characteristics are relevant for the likelihood of becoming a *sooner-than-intended* or an *unintended* parent, they do not explain the East–West disparity (H2). At the same time, however, we expect the effect of parity to be stronger in the East than in the West (H3). We assume that the geographic disparity can be largely, if not entirely, attributed to higher prevalence of uncertain fertility intentions in the East (H4) and to sociocultural factors: higher social pressure on having a child (H5) and higher anticipated costs of having a (further) child (H6) in the East. We do not assume any substantial East–West differences in the anticipated benefits (H7). Consequently, the East–West difference should weaken or disappear entirely once controlling for uncertainty of fertility intentions and sociocultural factors.

## Data and Methods

### Data

For our analysis, we use data from the first and second waves of the Generations and Gender Survey (GGS) (Gauthier et al., 2018) for Austria, Bulgaria, France, Hungary, Italy, and Poland.^3^ The GGS was carried out in the 2000s and 2010s, with either a three-year (Bulgaria^4^ and France) or a four-year (Austria, Hungary, Italy, and Poland) span between the waves. In Bulgaria, France, Italy, and Poland, questions about fertility intentions were posed to women and men aged 18–50; but in Austria and Hungary, the age range of the respondents was narrowed to 18–45 and 21–45, respectively. We therefore restrict our analysis to women and men aged 21 to 45 at wave 1 who stated their childbearing intentions and who had a child between waves 1 and 2. As it has been extensively documented that finding a new partner often triggers the intention to have a (further) child, we focus our main analysis on respondents with the same partner at both survey waves.^5^ Respondents are considered partnered if they are in a union, regardless of whether the union is coresidential or a LAT relationship.^6^ Our analytical sample comprises 1,175 female and 892 male panel respondents.

In the unrestricted sample, panel attrition ranges from 22% in Austria to 38% in Poland. Among respondents fulfilling the criteria of age and partnership status (partnered respondents only) and with known fertility intentions,^7^ the figures vary between 20% in Austria and 41% in Poland. As only one-fifth of the sample was re-interviewed in Italy, it is not possible to compute the attrition rates for that country (for details, see the documentation for Italy at http://www.ggp-i.org). Although attrition was quite high in some countries, it did not produce any substantial bias in any of the variables of interest (see Table 4ab in Appendix).

### Measures of Fertility Intentions and their Realisation

We focus on short-and long-term fertility intentions stated at wave 1.^8^ Short-term childbearing plans were captured by two questions: (1) *Do you yourself want to have a/another baby now?* (in France the question read: *Are you trying to have a baby now?*) and (2) *Do you intend to have a/another child during the next three years?* The possible answers to question (1) were *yes* and *no*. In case of question (2) they were *probably yes*, *definitely yes*, *probably not*, *definitely not*. In France, a fifth option was offered (*don’t know)*, whereas in Hungary, only three options were provided (*yes*, *no,* and *don’t know)*. The women and men who indicated that they did not intend to have a child in the near future (i.e. who answered *probably not*, *no,* or *definitely not* to question (1) and/or (2)) were asked a further question (3) about their long-term fertility intentions: *Supposing you do not have a/another child during the next three years, do you intend to have any (more) children at all?*^9^ The possible answers were specified in the same way as in question (2). For the descriptive analysis, we dichotomise the answers so that childbearing intentions are either positive or negative.^10^ In the multivariate analysis, however, we distinguish between certain (*definitely yes/definitely not*) and uncertain (*probably yes/probably not*) intentions, and thus exclude Hungary (see more details in the Sect. 4.3). Table 4b in Appendix shows the unweighted distribution of short-and long-term fertility intentions.

Based on the answers respondents gave at wave 1, we group them into three categories (see also Table 1):

- *intended parents*: declared at wave 1 the intention to have a child within the following three years, and had one by wave 2;
- *sooner-than-intended parents*: declared at wave 1 the intention to have a child at a later point in time than within the following three years, but had one by wave 2;
- *unintended parents*: declared at wave 1 that they did not intend to have a (further) child at any point in time, but had one by wave 2;

**Table 1:** Classification of respondents who had a child between waves 1 and 2, based on their fertility intentions at wave 1

Respondent group	Wave 1			Wave 2
	Q (1): Intention now	Q (2): Intention within three years	Q (3): Intention later than within three years	
Intended parents	Yes	DY/PY/Y	X	A child born between wave 1 & 2
	Yes	DN/PN/N		
	No/NA	DY/PY/Y		
Sooner-than-intended parents	No/NA	DN/PN/N/DK/NA	DY/PY/Y	
Unintended parents	No/NA	DN/PN/N/DK/NA	DN/PN/N	

The categories partially follow the terminology introduced by Spéder and Kapitány (2009). The abbreviations should be read as follows: *DY *Definitely yes, *PY* Probably yes, *Y* Yes, *DN *Definitely not, *PN* Probably not, *N* No, DK Don't know, NA No answer, *X* Not asked

Q (1): *Do you yourself want to have a/another baby now?* (in France: *Are you trying to have a baby now?)*

Q (2): *Do you intend to have a/another child during the next three years?*

Q (3): *Supposing you do not have a/another child during the next three years, do you intend to have any (more) children at all?*

We include only births that occurred up to three years (or 36 months) after wave 1. As the observation window in Bulgaria was only around 2.5 years, we classify pregnancies reported at wave 2 in Bulgaria as births.^11^ Throughout the study, we analyse whether any children were born between waves 1 and 2 (or, in the case of Bulgaria, were going to be born), without counting their actual numbers.

### Analytic Strategy

Our study is carried out separately for *unintended* and *sooner-than-intended* mothers and fathers. It has two parts: descriptive and multivariate. The descriptive analysis uses post-stratification weights to estimate the prevalence of births classified as unintended and sooner-than-intended. We first compute the proportions of these births among all births occurring between the two survey waves in each country. Then, we analyse the share of *unintended* and *sooner-than-intended* births by parity. For this purpose, we group the countries and distinguish between East and West Europe. The former comprises Bulgaria, Hungary, and Poland, whereas the latter includes Austria, France, and Italy. The country samples are re-weighted so that they are equally large for each country. Next, we check to what extent the characteristics of *intended, sooner-than-intended* and *unintended* parents in East and West differ from each other.

In the analysis, we include the following respondents’ characteristics: sex, age measured as a continuous variable, number of children (parity; 0, 1, and 2 +), education (university degree vs. secondary education and below), employment (employed vs. not employed), contraceptive use (yes or no), certainty of fertility intention (certain when the answer was “definitely” and uncertain when the answer was “probably”), the perceived social pressure on having a child, and the anticipated costs and benefits of having a child. The group of *employed* consists of respondents who were employed, self-employed (including farmers), or on maternity/paternity/parental leave. The sociocultural factors–the perceived social pressure to have a child, and the expected costs and benefits of having a child–are measured by indices constructed from 11 variables with the use of confirmatory factor analysis (CFA). Social pressure was captured by asking respondents whether they felt that their (a) parents, (b) relatives and (c) friends thought that they should have a(nother) child (see Table 5a in Appendix for exact question wordings). Possible answers to each of the three questions were: *strongly disagree, disagree, neither agree nor disagree, agree, strongly agree*. Costs and benefits were derived from respondents’ assessment of the effect they thought a child born within the following three years would have on: (d) the possibility to do what they want, (e) their employment opportunities, (f) their financial situation, (g) what people around them think of them, (h) the closeness between them and their partner/spouse, (i) certainty in their lives and (j) the closeness between them and their parents, (k) the care and security they may get in old age (see Table 5a in Appendix for details). Possible answers were: *much worse, worse, neither better nor worse, better, much better*. After recoding, the answers (see Table 5a), items (d)-(f) represent the costs of having a child, whereas items (g)-(k) are to be interpreted as benefits of having a child. Table 2 shows the sample characteristics.

**Table 2:** Panel sample characteristics, unweighted data

	Austria		France		Italy		Bulgaria		Hungary		Poland	
	Women	Men	Women	Men	Women	Men	Women	Men	Women	Men	Women	Men
*Classification of parents (response variables) *(%)												
Intentional parents	85	80	92	85	81	84	76	73	65	66	70	73
Sooner-than-intended parents	6	10	3	4	7	7	6	5	18	18	7	9
Unintended parents	8	10	4	10	11	9	19	21	17	16	23	19
*Explanatory variables *(%)												
Age (mean)	29.1	31.3	28.5	31.9	31.6	34.2	28.0	30.6	27.8	30.0	29.1	31.2
Parity												
0	44	50	46	41	34	41	34	25	41	44	30	30
1	36	33	35	37	47	46	54	63	37	31	47	51
2 +	20	17	19	22	19	13	13	12	22	26	24	20
Education												
Secondary and below	74	77	39	59	77	85	63	81	68	79	59	65
Tertiary	26	23	61	41	23	15	37	19	32	21	41	35
Missing	0	0	0	0	0	0	0	0	0	0	1	0
Employed	90	92	78	90	73	97	70	64	89	91	68	91
Using contraceptives	67	68	69	57	–	–	65	59	–	–	57	63
*Certainty of fertility intentions*												
Certain (definitely)	67	66	71	68	45	46	85	83	–	–	71	70
Uncertain (probably)	33	34	29	32	55	54	15	17	–	–	29	30
Social pressure (mean)	0.77	0.61	0.67	0.60	–	–	0.98	0.90	0.67	0.49	0.69	0.72
Missing (%)	0	0	0	0	–	–	0	1	0	0	0	0
Expected costs (mean)	− 0.24	− 0.34	− 0.38	− 0.52	−	–	− 0.11	− 0.28	− 0.22	− 0.35	0.01	− 0.17
Missing (%)	0	0	0	0	–	–	0	1	0	0	0	0
Expected benefits (mean)	0.07	0.09	0.12	0.21	–	–	0.23	0.26	0.14	0.18	0.10	0.07
Missing (%)	0	0	0	0	–	–	0	1	0	0	0	0
N (total)	233	163	173	116	231	176	127	75	222	180	189	182
*Survey year*												
Wave 1	2008/09		2005		2003		2004		2004/05		2010/11	
Wave 2	2012/13		2008		2007		2007		2008/09		2014/15	

All explanatory variables were measured at wave 1. The variables *social pressure on having a child* and *expected costs and benefits of having a (further) child* are standardised variables, with mean equal 0 and standard deviation equal 1 (when computed for the entire panel sample).

In the multivariate analysis, we apply logistic models to identify the risk factors of having a birth that can be classified as sooner-than-intended or unintended: we estimate the odds of a birth being (a) *sooner-than-intended* or (b) *unintended* as opposed to being *intended* (separately for a) and b)).^12^ We apply a step-wise procedure in which we test how the individual characteristics affect the relationship between the probability of becoming a *sooner-than-intended* or *unintended* parent and the region of Europe (East–West). Thus, each model includes a macrolevel dummy variable that distinguishes between the Eastern and the Western part of Europe. Beyond that, we control for an increasing number of characteristics, starting from sex, age (and age squared), parity, education, employment and contraceptive use in model one (M1). In M2, we interact parity with the East–West dummy as we assume that the effect of parity differs between East and West. Next, in M3, we control also for uncertainty of fertility intentions. In the last three steps, we add social pressure (M4) and the anticipated benefits (M5) and costs (M6) of having a child. All covariates in all six models are interacted with respondent’s sex, so that the estimates for women and men can be directly compared as coming from the same models.

The information on contraceptive use is not available for Hungary and Italy. Moreover, the (un) certainty of fertility intentions was not assessed in Hungary as respondents could answer only *yes* or *no* to the questions about fertility intentions. In Italy, social pressure on having a (further) child was asked in a different way than in the standard questionnaire, and so we could not compute the sociocultural indicators for this country. For these reasons, we run all models for only four countries: two from the West (Austria and France) and two from the East (Bulgaria and Poland).

## Results

### Descriptive Analysis

#### Prevalence of *unintended* and *sooner-than-intended* Parents

**Fig. 1:**
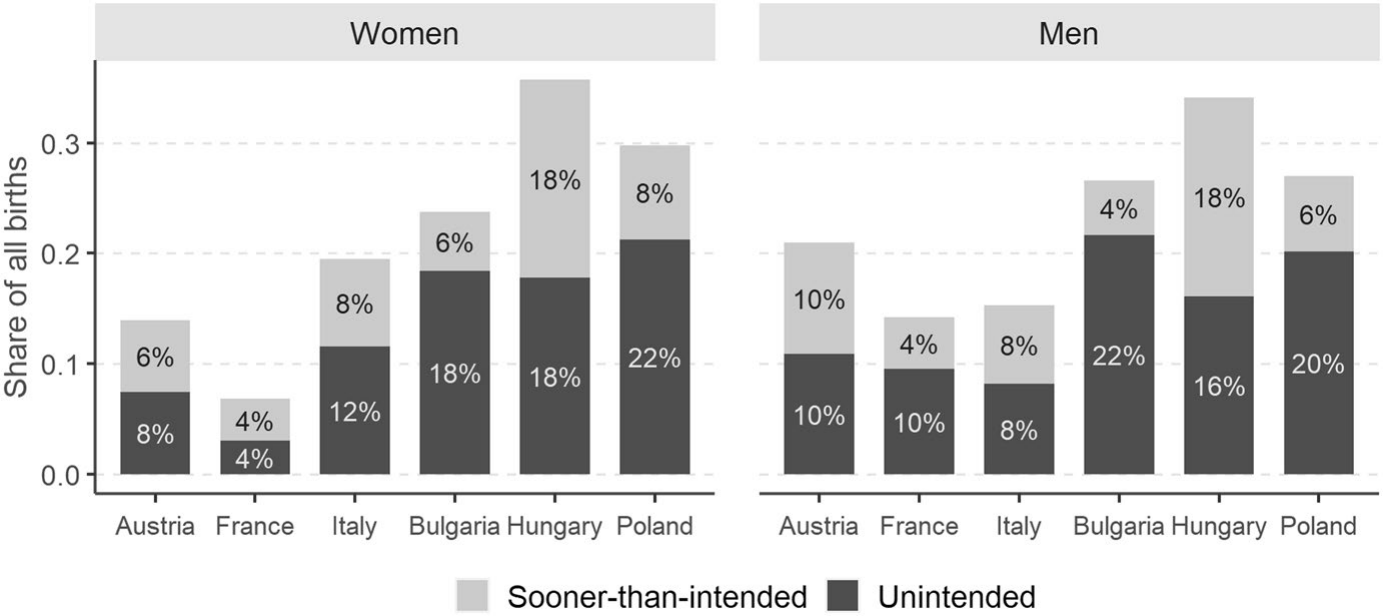
Share of *sooner-than-intended* and *unintended* parents among all respondents who had a child between waves 1 and 2, by country and sex, weighted data, respondents with the same partner at both survey waves. *Source: Authors’ computations,* *N* = *2,067*

Of all respondents who had a child between waves 1 and 2, between 4% (among women in France) and 22% (among men in Bulgaria) were *unintended* parents (Fig. 1).^13^ The share of *sooner-than-intended* parents was usually lower, with lowest values in France and highest values in Hungary. Thus, between 8% (among women in France) and 36% (among women in Hungary) of parents were either *unintended* or *sooner-than-intended*. Among both women and men, there was a clear East–West divide in the shares of *unintended* parents: in Bulgaria, Hungary, and Poland they varied between 16 and 22%, whereas in Austria, France, and Italy they ranged from 4 to 12%. For the share of *sooner-than-intended* parents, we find no consistent geographic variation.^14^

**Fig. 2:**
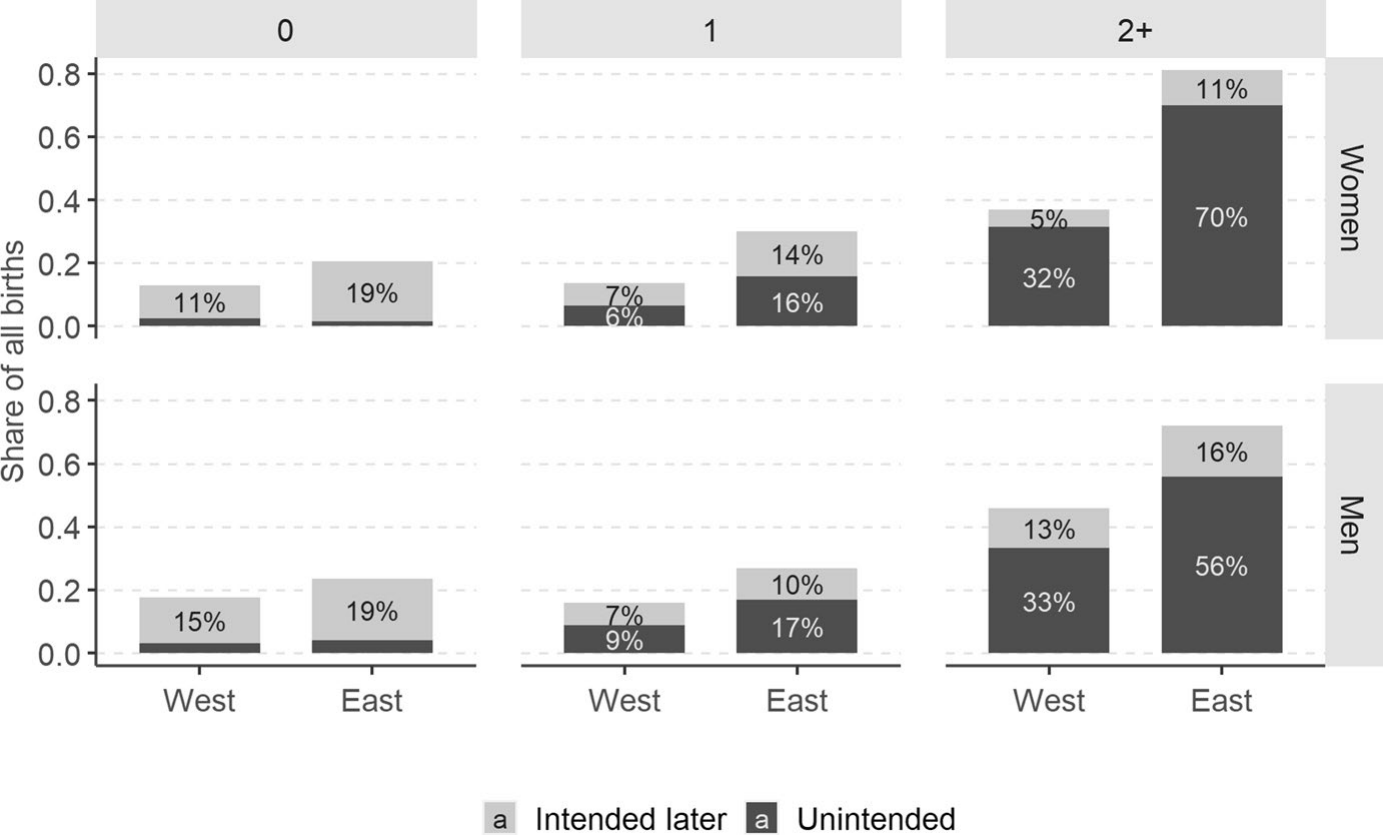
Share of *sooner-than-intended* and *unintended* parents among all respondents who had a child, by parity, region and sex, weighted data, respondents with the same partner at both survey waves. *Source: Authors’ computations, N* = *2,067*

Breaking down the analysis by parity shows that the East–West divide is driven primarily by respondents who at wave 1 had two or more children (Fig. 2). In the East, 66% and 54% of births to mothers and fathers, respectively, of at least two children were *unintended*; the corresponding numbers in the West were 27% and 33%. At the same time, in both parts of Europe high-order births (i.e. births to parents who already have at least two children) constitute a lion’s share of all unintended births: almost two-thirds among women and a half among men (Fig. 9 in Appendix). The geographic pattern can also be observed for *sooner-than-intended* high-order births: their share among women with two or more children reached 12% in the East and 6% in the West. For men, the figures amounted to 18% and 6%, respectively.

Among one-child parents, the share of *unintended* births is dramatically lower whereas that of *sooner-than-intended* is at a similar level. Nevertheless, the East–West divide remains sharp in the share of both types of births. It disappears only for first-time parents (i.e. respondents who were childless at wave 1), of whom less than 10% were *sooner-than-intended* and only up to 3% were *unintended*.

#### Characteristics of *sooner-than-intended* and *unintended* Parents in East and West

Table 3 summarises the descriptive characteristics of *sooner-than-intended* and *unintended* parents and compares them to those of *intended* parents in Eastern and Western Europe. In line with previous studies, we find the *sooner-than-intended* and *unintended* parents to be younger and older, respectively, than the *intended* parents. The sex distribution also follows our expectations but only in the West where there are more men than women among the *sooner-than-intended* and *unintended* parents. In the East, women prevail in both groups. Contraceptives were more often used at wave 1 by the *sooner-than-intended* and *unintended* parents than by *intended* ones. The figures are consistently lower in the East than in the West, but the difference is not dramatic. The *intended* parents have more often university education than the two other groups and the gap is wider in the East. They are also more likely to be employed but only in the East. In the West, there are no differences in employment between the three groups of parents.

**Table 3:** Characteristics of *intended, sooner-than-intended* and *unintended* parents by region, weighted data, respondents with the same partner at both survey waves

	Intended		Sooner-than-intended		Unintended	
	West	East	West	East	West	East
Women (%)	54	55	43	59	35	55
Age (mean)	29.8	29.5	27.7	26.9	33.5	32.5
*Parity distribution*						
Childless (%)	50	41	50	17	15	1
With 1 child (%)	37	56	32	46	34	48
With 2 + children (%)	13	4	18	37	52	50
University education (%)	39	40	27	19	28	12
Employed (%)	86	78	87	50	87	65
Using contraceptives (%)	63	58	79	78	78	66
Uncertain fertility intentions (%)	30	14	52	63	49	36
Social pressure (mean)	0.75	1.09	0.57	0.49	0.05	0.09
Expected costs (mean)	− 0.41	− 0.25	− 0.21	0.24	− 0.07	0.18
Expected benefits (mean)	0.15	0.27	0.14	0.04	− 0.03	− 0.17

As some characteristics are not measured in Hungary and/or Italy, for the sake of consistency these two countries are excluded. *N* = 1,257.

Social pressure as well as the expected costs and benefits are standardised variables with mean at 0 and standard deviation equal to 1. Interpretation: 0.75 is to be interpreted as “0.75 above the average” and − 0.41 is equivalent to “0.41 standard deviation below the average”.

Table 6 in Appendix presents numbers for all six countries

The Eastern and Western Europeans differ substantially with respect to parity distributions. In the West, the parity distribution among *intended* and *sooner-than-intended* parents is very similar, with half of births occurring among childless couples, around one-third among parents of one child and about one-seventh among parents with two or more children. Among unintended parents, the proportions flip: childless respondents are the smallest group and parents of at least two children are by far the biggest group. In the East, *intended* and *sooner-than-intended* parents comprise mainly couples who already had one child at wave 1 (56% and 46%, respectively; Table 3). The former include a large fraction of childless respondents and a negligible number of parents with two or more children. Among *sooner-than-intended* parents, the share of respondents with at least two children is over twice as high as that of childless respondents. The Eastern European *unintended* parents consist almost entirely and equally of respondents with one and two or more children.

The degree of uncertainty in childbearing intentions is highest among *sooner-than-intended* parents, with a majority of respondents being uncertain about their fertility plans. Among the *unintended* parents, the numbers do not substantially change in the West but they almost halve in the East. The *intended* parents have the most certain intentions, much more so in the East than in the West.

**Fig. 3:**
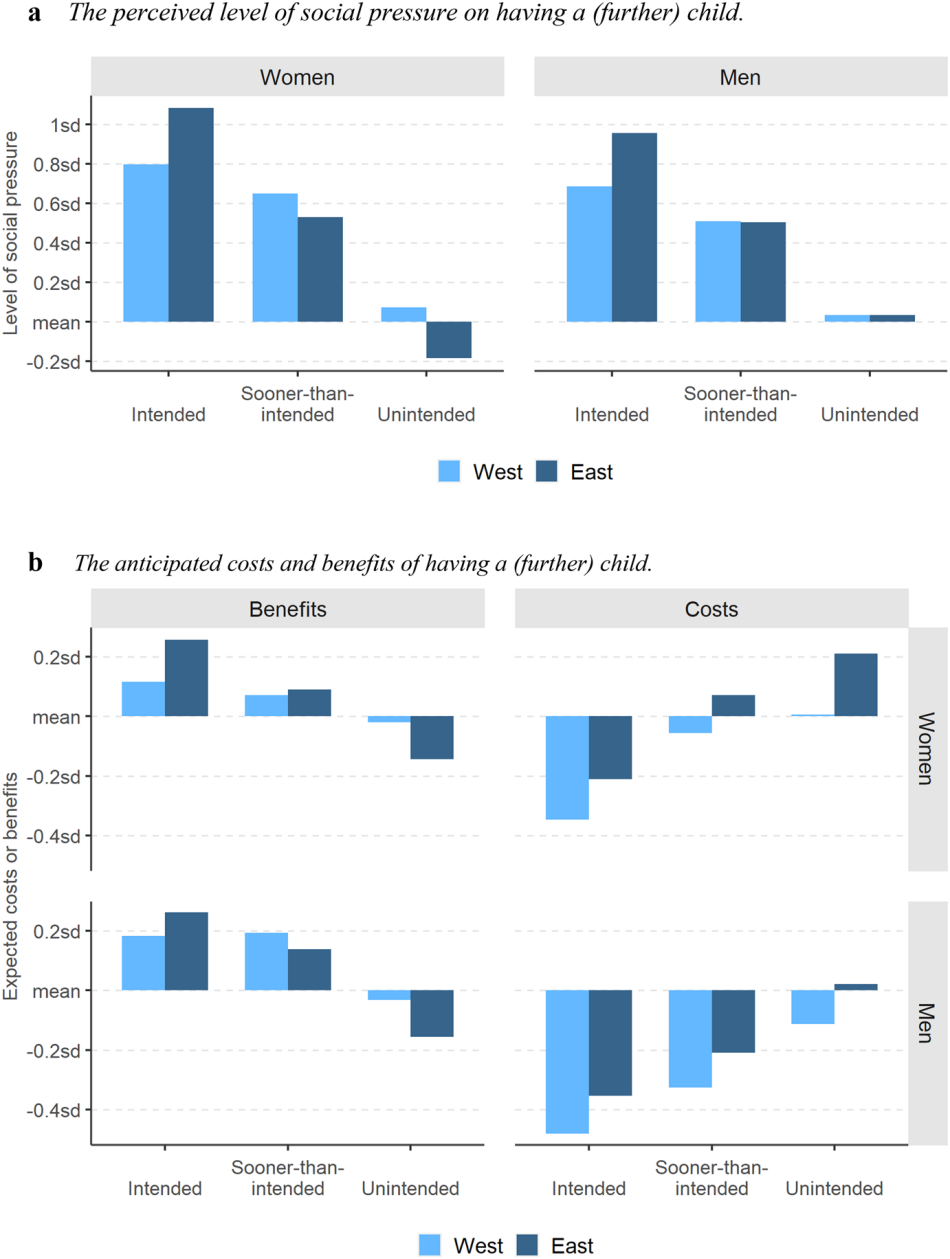
Sociocultural factors among *intended, sooner-than-intended* and *unintended* parents, by region and sex, weighted data, respondents with the same partner at both survey waves. **a** *The perceived level of social pressure on having a (further) child.* **b** The anticipated costs and benefits of having a (further) child.* Note* The indices of expected costs and benefits of having a (further) child are standardised variables with mean at 0 and standard deviation equal to 1 (when computed for the entire panel sample). Interpretation: as for Table 3. Italy excluded. *Source: Authors’ computations, N* = *1,659*

The perceived level of social pressure on having a (further) child is, as expected, highest for *intended* parents and lowest for the *unintended* ones. This holds for both women and men in the Eastern as well as Western part of Europe (see Fig. 3a). *Intended* parents feel more pressure in the East than in the West. However, among *sooner-than-intended* and *unintended* parents the geographic pattern reverses for women (the pressure becomes higher in the West than in the East) and disappears for men. The anticipated costs and benefits of having a (further) child also go in line with our expectations, both for women and men (Fig. 3b). They are lowest (costs) and highest (benefits) among *intended* parents and highest and lowest, respectively, among *unintended* parents. Western Europeans expect systematically lower costs than Eastern Europeans. However, this pattern does not entirely correspond with the assessment of the benefits. Compared to the West, respondents in the East indeed anticipate lower benefits, but only if they are classified as unintended parents. Among *sooner-than-intended* mothers and fathers, there is no substantial East–West difference, whereas the *intended* parents expect higher benefits in the East than in the West.

### Multivariate Analysis

#### East–West Differences in the Probability of Becoming a *sooner-than-intended* or an *unintended* Rather than an *Intended* Parent

**Fig. 4:**
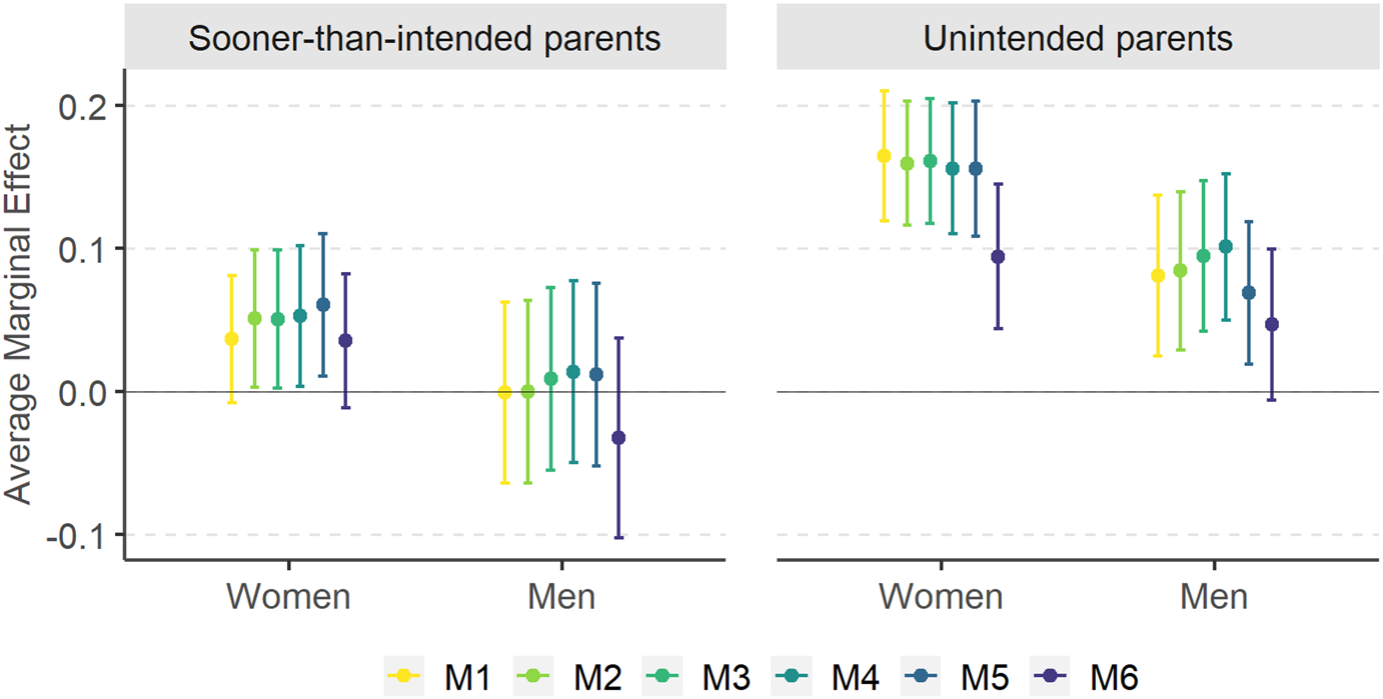
*Sooner-than-intended* and *unintended* parents in comparison with *intended* parents: average marginal effects of the East (vs. reference category: West) in models M1–M6 together with 95% confidence intervals.* Note* Average marginal effects (AMEs) expressed on the scale of the response variable (as changes in probabilities) from logistic models M1–M6 estimated jointly for women and men. Covariates included in consecutive models (all terms interacted with sex): M1: east–west + age + age^2^ + parity + education + employment + contraceptive use, M2: as in M1 + parity x east–west, M3: as in M2 + uncertainty of fertility intentions, M4: as in M3 + social pressure, M5: as in M4 + expected benefits, M6: as in M5 + expected costs. Countries included: Austria, France, Bulgaria and Poland. For full model specifications, see Table 7 in the Appendix

For each pair of models predicting the log-odds of being (a) a *sooner-than-intended* and (b) an *unintended* parent rather than an *intended* parent, we compute six specifications. M1 is the baseline model, which we iteratively expand by additional predictors (M2 to M6). Figure 4 presents the effects of the East vs. the West (reference category) as average marginal effects (AMEs) calculated from models M1 to M6. The AMEs denote an average change in the probability of being a *sooner-than-intended* (left-hand panel) or an *unintended* parent (right-hand panel) rather than an *intended* parent (reference category) attributed to living in the East rather than in the West. The probability changes are expressed in percentage points (p.p.). The full model specifications and their goodness-of-fit statistics are shown in Table 7 in the Appendix.

The results demonstrate that the East–West disparity in the probability of becoming an *unintended* rather than an *intended* parent is largely driven by the different assessment of the costs of having a (further) child in East and West. The results from the first five models (M1–M5) suggest that women in the East are about 16 p.p. more likely to become an *unintended* rather than an *intended* mother. For men, the figure oscillates between 7 and 10 p.p. However, once we control for the anticipated costs of having a child (M6), the effect of the East drops to 9 p.p. for women^15^ and becomes statistically not significant for men (at the 0.05 significance level). When it comes to the likelihood of being a *sooner-than-intended* rather than an *intended* parent, we find no East–West differences among men but among women the probability is higher in the East by about 5 p.p. in models M2 to M5. The effect appears when including the interaction between parity and the East–West dummy variable but disappears when controlling for the expected costs of a child (M6). This means that parity has a different effect on the probability of becoming a *sooner-than-intended* rather than an *intended* mother in the East than in the West. When analysing the average marginal effects of parity calculated from model M2 (Fig. 10 in Appendix), we see that in the West parity does not play any role but in post-socialist countries women who already have at least two children are much more likely to have a further birth classified as sooner than intended. This makes the likelihood of being a *sooner-than-intended* mother higher in the East than in the West. However, this effect disappears once we control for the expected costs of having a child, which suggests that Eastern European women with at least two children become *sooner-than-intended* mothers more often than their Western counterparts because they assess the costs of having a further child differently.

**Fig. 5:**
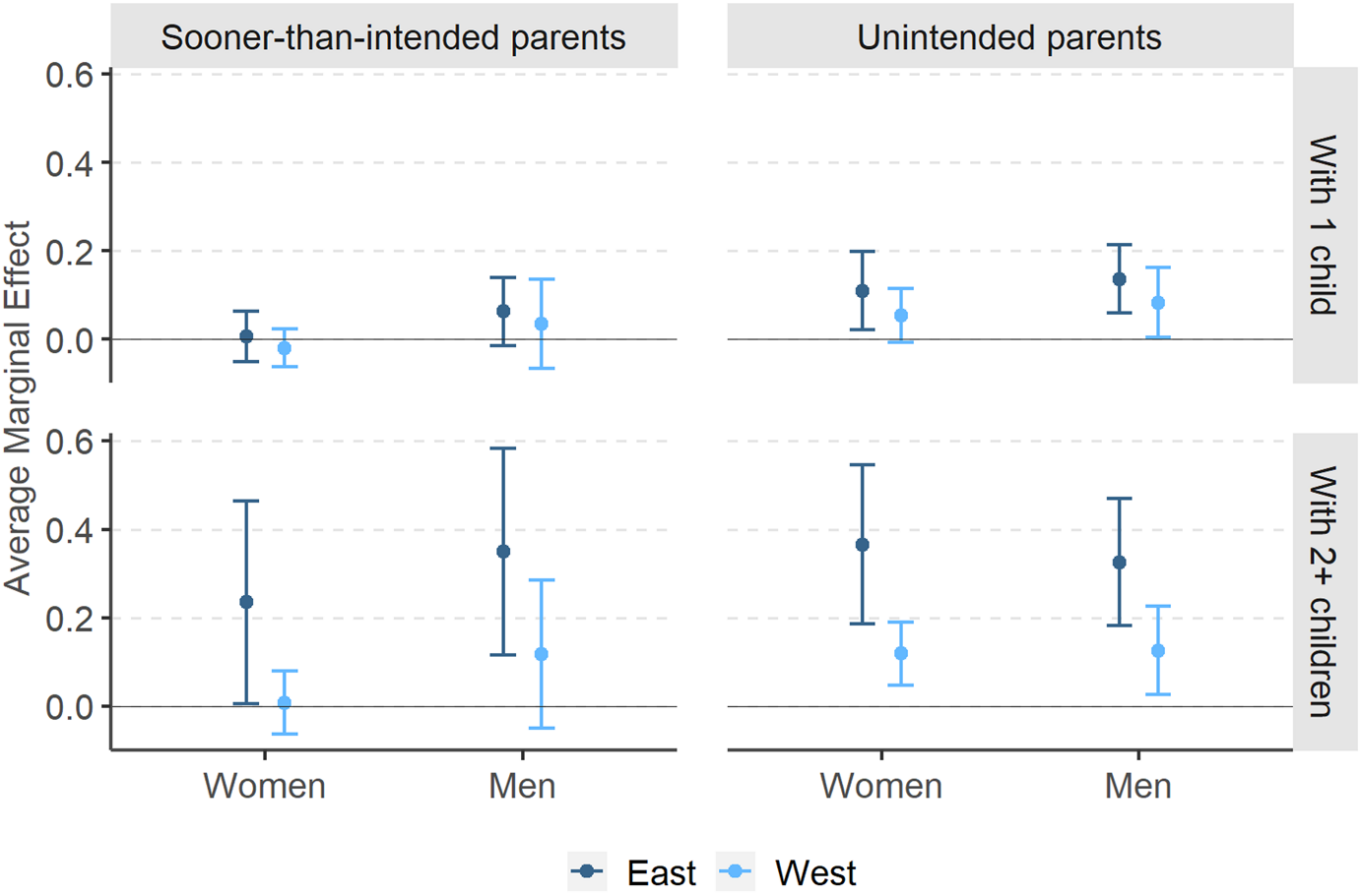
*Sooner-than-intended* and *unintended* parents in comparison with *intended* parents: average marginal effects of parity in East and West together with 95% confidence intervals (reference category: childless respondents). Note: Average marginal effects (AMEs) expressed on the scale of the response variable (as changes in probabilities) from joint logistic M6 models for women and men. Countries included: Austria, France, Bulgaria and Poland. For full model specification, see Table 7 in Appendix

At the same time, the East–West difference in the effect of parity remains even when controlling for the anticipated costs of a child in model M6 (Fig. 5). Compared to childless respondents, Eastern European mothers and fathers of at least two children are between 23 p.p. (women) and 35 p.p. (men) more likely to have a child *sooner than intended*, whereas in the West their probability does not differ from that for childless respondents or one-child parents. In case of the *unintended* parents, parity plays an important role in both Western and Eastern Europe. Having already one child rather than none increases the probability of experiencing an *unintended* birth by over 10 p.p. for women and men in the East and by nearly 10 p.p. for men in the West (for Western European women the effect is not significant). For parents of at least two children, the likelihood rises by over 10 p.p. in the West and by over 30 p.p. in the East for both sexes.

The results presented in Figs. 4 and 5 support five out of seven of our hypotheses. The likelihood of experiencing a *sooner-than-intended* or an *unintended* birth rather than an *intended* one is higher in post-socialist than in Western-European countries (H1), albeit in case of *sooner-than-intended* parents the gap is overall much weaker and appears only when controlling for parity and only for women. As predicted by H2, the East–West divide does not depend on any sociodemographic characteristic or contraceptive use (M1 and M2), at least when controlling for the anticipated costs. The effect of parity operates much more strongly in the post-socialist countries than in Western Europe, which supports H3. At odds with H4 and H5, the geographical disparity is related neither to the uncertainty of childbearing intentions (model M3 does not change the effect of the East as compared to model M2) nor to the perceived level of social pressure (no difference between model M4 and model M3). The East–West divide is either largely or entirely driven by a different assessment of the costs of having a (further) child (H6), whereas the anticipated benefits do not play any role for the East–West differences (H7).

#### Risk Factors of Becoming a *sooner-than-intended* or an *unintended* Rather than an *Intended* Parent

**Fig. 6:**
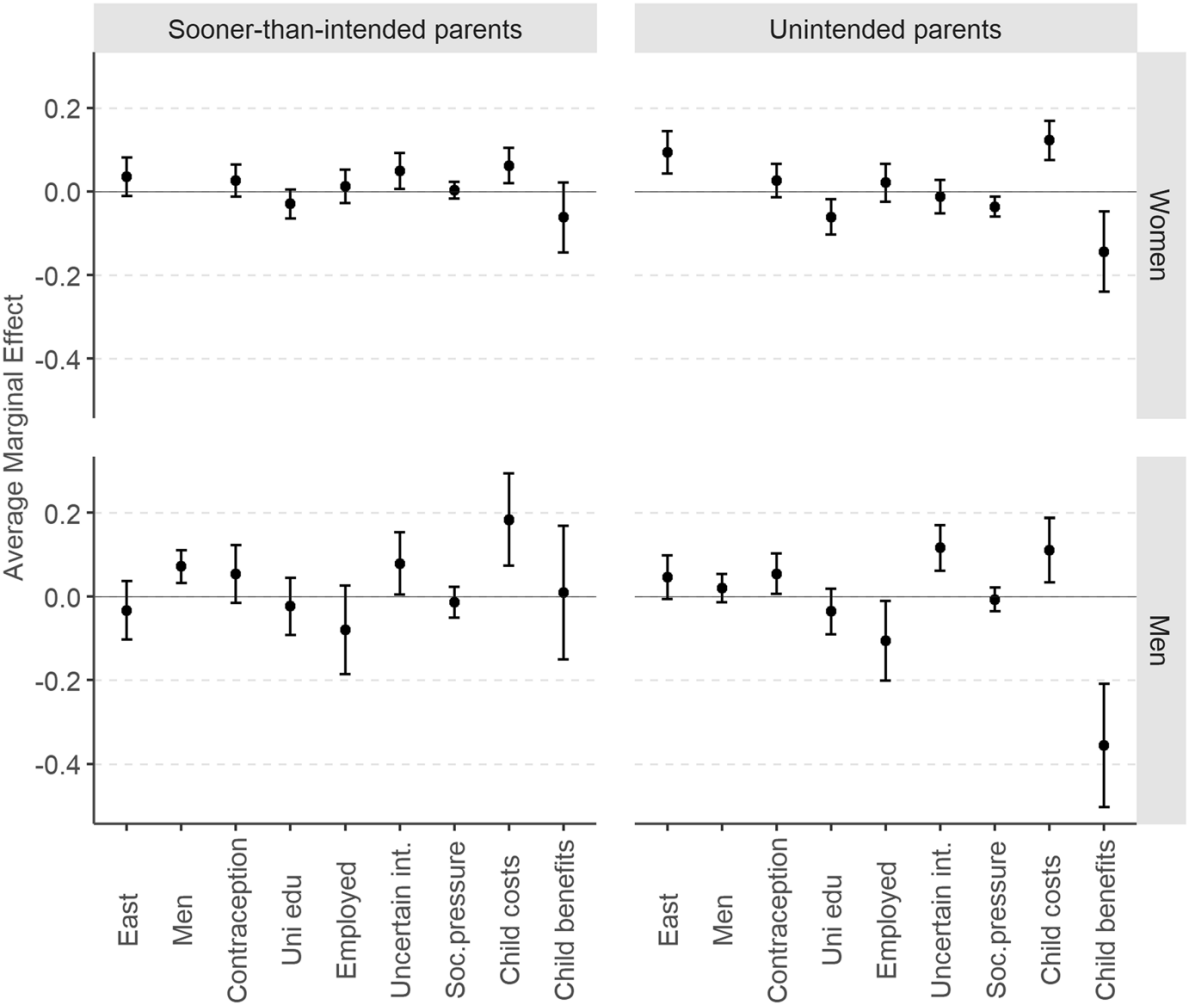
*Sooner-than-intended* and *unintended* parents in comparison with *intended* parents: average marginal effects together with 95% confidence intervals. Note: Average marginal effects (AMEs) expressed on the scale of the response variable (as changes in probabilities) from joint logistic M6 models for women and men. For the variables social pressure, child costs and benefits, AME expresses a change in the probability in response to an increase by one standard deviation. Reference categories of the categorical variables: West, women, no contraception, secondary education or lower, not employed, certain about fertility intentions. Countries included: Austria, France, Bulgaria and Poland. For full model specification, see Table 7 in the Appendix

**Fig. 9:**
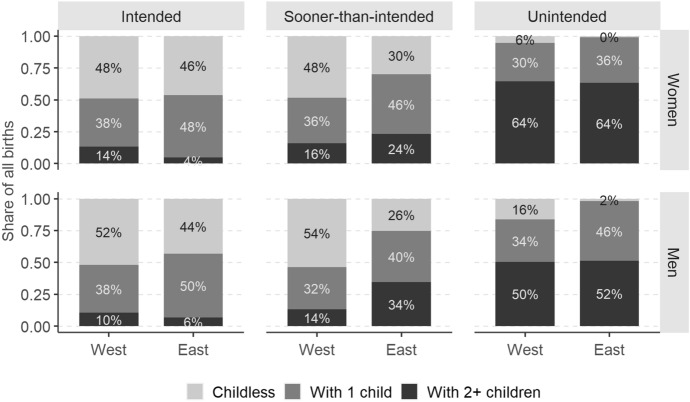
Parity distribution among *unintended, sooner-than-intended*, and *intended* parents, by country and sex, weighted data. Source: Authors’ computations, N = 2,067

**Fig. 10:**
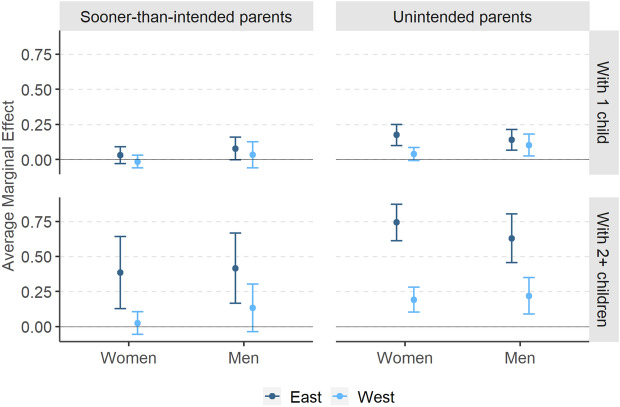
*Sooner-than-intended* and *unintended* parents in comparison with intended parents: average marginal effects of parity in East and West computed from model M3, together with 95% confidence intervals

**Fig. 11:**
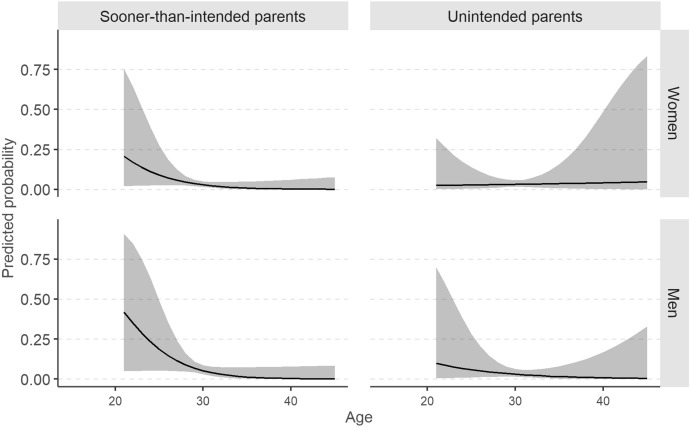
Predicted probabilities of being *sooner-than-intended* and *unintended* parents rather than *intended* parents at different age, women and men, together with 95% confidence intervals

Our results largely corroborate previous findings on the sociodemographic factors associated with *sooner-than-intended* or *unintended* parenthood as opposed to *intended* parenthood. Figure 6 shows the average marginal effects of sociodemographic variables computed from the full model (M6). The role of parity has been already discussed (and demonstrated in Fig. 5) and is thus omitted from the graph. The effect of age is shown separately as predicted probabilities (Fig. 11) to assess the effect of both age terms included in the model (age and age squared).

Generally, there are only a few characteristics that distinguish *sooner-than-intended* from *intended* parents. The probability to become the former rather than the latter rises for men (by 7 p.p. as compared to women), for respondents with uncertain childbearing intentions (by about 5 p.p. among women and 8 p.p. among men compared to women and men, respectively, who were certain about their childbearing plans), for respondents anticipating higher costs of having a child (increase by 6 p.p. among women and 18 p.p. among men per one standard deviation) and for respondents, particularly men, in their early 20s (Fig. 11).

In case of *unintended* parents, many covariates affect differently women and men. Among women, the likelihood of experiencing an *unintended* rather than an *intended* birth decreases with education (it is 6 p.p. lower for university diploma holders than for those with at most secondary education), the perceived level of social pressure (by 4 p.p. per one standard deviation) and the expected benefits of having a child (by 13 p.p. per one standard deviation), while increasing with the anticipated costs of having a child (by 14 p.p. per one standard deviation). Among men, the probability is higher for those who at wave 1 used contraceptives (by 5 p.p. compared to those who did not use contraceptives), were not employed (by 11 p.p. compared to those employed), were uncertain about their childbearing plans (by 12 p.p. compared to those who were certain) and assessed the costs as high and the benefits as low (increase by 11 p.p. and 35 p.p. per one standard deviation above and below the mean, respectively).

## Discussion

This paper has provided evidence on women and men classified by the prospective approach as unintended and sooner-than-intended parents in Europe. Our results show that in our study population (non-teenage respondents with a stable partner who had a child between the first and second survey wave), between around 10% (non-post-socialist countries) and 30% (post-socialist countries) experienced an *unintended* or a *sooner-than-intended* birth. The share of *unintended* parents is clearly higher in Bulgaria, Hungary and Poland than in Austria, France and Italy. However, the East–West divide weakens for women and disappears for men once we control for the anticipated costs of having a child.

We find that the anticipated costs and benefits of having a child are among the main factors associated with experiencing a *sooner-than-intended* or an *unintended* birth rather than an *intended* one. Respondents who intended a child at wave 1 and realised that intention by wave 2 expected the highest benefits and the lowest costs whereas the *unintended* parents assessed the benefits as lowest and the costs as highest. Moreover, in all three groups, Eastern Europeans expected systematically higher costs than respondents from the West. Consequently, the East–West divide in the share of *unintended* parents turned out partly spurious: to a large extent, it simply reflects the East–West differences in the assessment of the costs of having a (further) child.

Why are the expected costs of having a child higher in post-socialist countries? Answering this question is beyond the scope of our analysis, but, as already pointed out in an earlier part of the text, the most probable explanation is that an average family faces tougher socioeconomic conditions in the East than in the West, such as: cramped housing conditions, hardly any part-time jobs, few childcare places for under-three-year-olds, little support from the state (the GGS was conducted before large-scale family social programmes were introduced in Poland and Hungary; Sobotka et al., 2019) and relatively low salaries. The labour market tends to have relatively weak protections for employees while the low unemployment benefit levels provide only a thin safety cushion in case of job loss (European Commission, 2018).

The perceived level of social pressure on having a child also shows clear intention-related and geographic patterns in the descriptive analysis but once included in the multivariate analysis it barely has any effect. One potential explanation for this apparent inconsistency is the ambiguous role of social pressure in reproductive decision-making. On the one hand, past research showed a positive relationship between social pressure and short-term childbearing intentions (Balbo & Mills, 2011; Billari et al., 2009) and, mostly through intentions, childbearing (Kuhnt & Trappe, 2016). Our study corroborates these results: the *intended* parents feel the strongest pressure and the *unintended* parents the weakest. On the other hand, however, we find that among *intended* parents the figures are higher in the East where the realisation of the intention to have a child is known to be lower than in the West. Among *sooner-than-intended* and *unintended* parents, the perceived social pressure is either lower in the East (women) or does not differ between the two regions (men). Thus, depending on the group, the figures are either similar in both regions or higher or lower in the East than in the West, and for this reason including social pressure in the model does not change the East–West differences in the probability of experiencing an *unintended* birth rather than an *intended* birth. Moreover, these results indicate that high levels of social pressure in the East may “inflate” the intention to have a child and in this way contribute to the lower realisation of fertility intentions in post-socialist countries. Analysing more closely the role of social pressure in the realisation of fertility intentions and their geographical differences should certainly be included in future studies. It may also add another layer to the argument of social anomie (Spéder & Kapitány, 2014).

Our finding that parity two and higher had a strong and positive effect on the likelihood of experiencing an *unintended* or, only in the East, a *sooner-than-intended* birth rather than an *intended* birth is also in line with previous results (D’Angelo et al., 2004; Dutta et al., 2015; Exavery et al., 2014). This pattern may be seen as a manifestation of the two-child norm. It has been shown that most women and men regard two as the ideal number of children (Sobotka & Beaujouan, 2014). This universally strong norm may make parents of two children more reluctant to explicitly admit that they would actually like to have more children. With parents facing rising expectations about the quality of parenting on the one hand, and growing pressure to successfully balance professional career and family life on the other, they often find it difficult to choose to have an additional child. As well as presenting practical challenges for most parents, having a large family is not very socially accepted (Lück & Bujard, 2018; Van Bavel et al., 2018). Consequently, it is possible that individuals who would like to have more children, but feel they lack the resources or the support to do so, might declare that they do not intend to have an additional child, and yet not be fully committed to that intention. Our observation that the effects of parity two and higher were much stronger in the East than in the West (and, in case of *sooner-than-intended* parents, statistically significant only in the East) only adds to the credibility of the two-child norm argument. On the one hand, the dominance of the two-child family model–which results in part from relatively low rates of progression to third birth–has been very strong in the East for decades, including during the era of state socialism. On the other hand, after 1989, the aspirations of middle-class parents for their children rose sharply in the East. But because families in the East still have more modest resources than their counterparts in the West, it can be more difficult for parents in the East to choose to have a third or further child (Brzozowska et al., 2017).

Do *sooner-than-intended* and *unintended* parents show traces of a negative selection process typical of women who experience a mistimed or unwanted pregnancy? In the East, *sooner-than-intended* parents are much more likely to already have two or more children but, as discussed in the previous paragraph, this may have nothing to do with a negative selection. Among *unintended* parents in East and West, we find an overrepresentation of women who have no university education and men who are not employed as well as mothers and fathers of at least two children. The evidence is insufficient to draw conclusions about intendedness of the births based on the sociodemographic characteristics of the three groups of parents.

The inability to answer the question of whether the *sooner-than-intended* and *unintended* births were in fact mistimed and unwanted is one of the main weaknesses of our study. The sample size did not allow us to test, e.g. the role of formalising a union by getting married, known to substantially increase the chances of a birth being classified as unintended by the prospective approach, but as wanted by the retrospective method (Rackin & Morgan, 2018), or the role of a job loss or the safety net offered to the unemployed in different countries or regions. Furthermore, it is important to keep in mind that our results do not account for induced abortions. We are aware that by not accounting for abortions, we may have underestimated the prevalence of *sooner-than-intended* and, especially, *unintended* births. This might be particularly problematic in the case of the post-socialist countries, as at the time the second survey wave was conducted the abortion ratios were substantially higher in countries like Bulgaria and Hungary than in France or Italy (WHO Regional Office for Europe, 2019; Austria does not collect data on induced abortions). We tried to assess the potential bias by comparing the “*unintended* and *sooner-than-intended* birth rates”, i.e. the number of *unintended* and *sooner-than-intended* births in relation to the number of respondents who at wave 1 did not intend to have a child (at all or within the following three years) across the three post-socialist countries. The figures for Poland did not differ substantially from those for Hungary and Bulgaria, although the Polish abortion law is very strict: i.e. at the time the GGS was conducted, it allowed for a legal termination of pregnancy only in cases of incest, rape, severe damage to the foetus, or danger to the mother’s life. There are no reliable estimates of the number of abortions performed on Polish women, but given the high cost of obtaining an abortion either on the black market or in one of the neighbouring countries, we can assume that the abortion ratios are much lower in Poland than in Hungary or Bulgaria. Thus, our finding that the “*unintended* and *sooner-than-intended* birth rates” were not higher in Poland than in Hungary and Bulgaria indicates that the bias of our results is not large.

Our study offers three methodological recommendations for improving cross-national panel surveys designed to examine childbearing intentions that would make it possible to use these surveys to analyse childbearing intendedness. First, in line with Gauthier et al. (2018), we argue that a cross-country survey must have one (standard) questionnaire implemented exactly in the same way in all participating countries if it aims to provide data that are cross-nationally comparable. We were able to carry out our analysis in full only on four out of 14 countries that fielded two waves of the GGS. However, even with these measures adopted, the data we use are not fully comparable, as Brzozowska and Beaujouan have convincingly demonstrated in their recent paper (2020). Second, analysing such complex concepts as fertility intentions and intendedness requires measurement that reflects their continuous rather than dichotomous character and their embeddedness in norms, values and attitudes. The significant effect of certainty of fertility intentions in our analysis clearly demonstrates that childbearing intentions should be measured at least on a four-point scale (*definitely not—probably not–probably yes–definitely yes*) or, preferably, a longer one (e.g. recently, a 10-point scale of fertility intentions has been successfully used; Guetto et al., 2020; Vignoli, 2019). The importance of the anticipated costs and benefits of having a child as well as the possibly contradictive role of social pressure shows the potential that indicators of norms, values and attitudes have for studying childbearing intentions, when transformed into composite continuous indices. In the future fertility and family surveys, we would welcome more detailed theory-guided questions about e.g. social pressure on and subjective costs and benefits of having children (see for instance Bein et al., 2020), social anomie and, more generally, the perceived uncertainty and the way respondents see their future and how they think about it.

Third, the assessment of birth intendedness would be much more accurate if, at the second survey wave, the parents of children born between the waves had been asked whether the births were wanted and came at the right time. Adding this one question to the questionnaire would combine the prospective and the retrospective approach, enabling researchers to carry out comparative analyses not only on childbearing intentions, but on childbearing intendedness. Such a strategy was successfully implemented in the French GGS, allowing for insightful analyses of changes in childbearing intendedness over time (Régnier-Loilier & Sebille, 2017).

## Figures and Tables

**Fig. 7:**
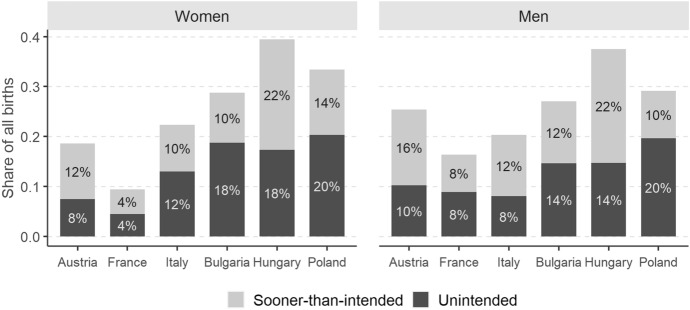
Share of *sooner-than-intended* and *unintended* parents among all respondents who had a child between waves 1 and 2, by country and sex, weighted data. *Source:* Authors’ computations, *N* = 2,680. Respondents with and without a partner at wave 1

**Fig. 8:**
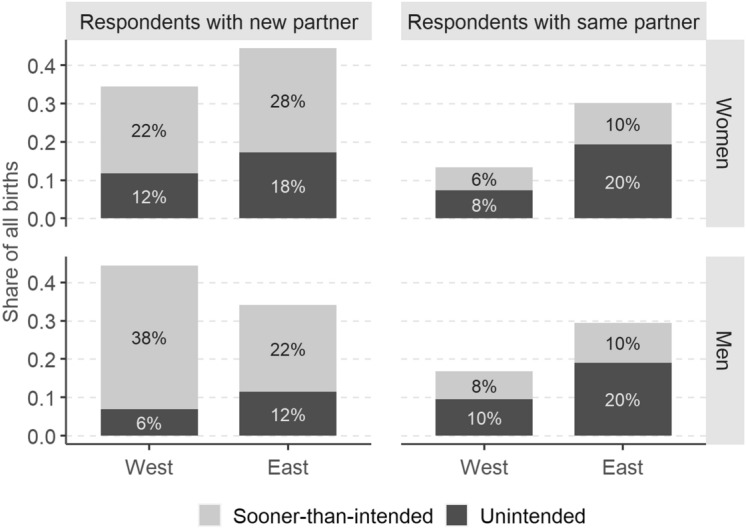
Share of *sooner-than-intended* and *unintended* parents among all respondents who had a child between waves 1 and 2, by change in partnership status, country and sex, weighted data. *Source*: Authors’ computations, *N* = 2,613. Respondents with a new partner include those who were single at wave 1 and partnered at wave 2 as well as those who changed partners between the survey waves. Respondents partnered at wave 1 and single at 2 were excluded

## Appendix

.

**Table 4:** Assessing the attrition bias

	Austria	Bulgaria	France	Hungary	Italy	Poland
a) Distribution of respondents by their relevant characteristics in the sample of wave 1 and in the panel sample (in %), unweighted data						
*Wave* 1						
Women (%)	61	61	58	56	60	58
Age (mean)	34.07	34.24	33.98	33.24	36.33	33.91
Parity						
Childless (%)	34	14	29	27	19	19
With 1 child (%)	23	34	19	23	27	31
With 2 + children (%)	43	51	52	49	54	50
University education (%)	21	23	39	19	13	31
Employed (%)	85	72	80	85	74	77
Using contraceptives (%)	78	74	84	–	–	70
Uncertain fertility intentions (%)	23	12	23	–	41	32
Social pressure (mean)	0.08	0.02	− 0.14	− 0.19	–	0.16
Effects on own life (mean)	− 0.01	0.02	− 0.02	0.02	–	− 0.02
Effects on relations (mean)	− 0.05	0.10	− 0.10	− 0.10	–	0.08
N	3,120	4,955	2,883	4,100	3,530	5,155
*Panel sample*						
Women (%)	60	61	58	58	60	61
Age (mean)	34.17	34.50	34.38	33.30	36.33	34.60
Parity						
Childless (%)	34	13	27	26	19	15
With 1 child (%)	22	33	18	23	27	29
With 2 + children (%)	44	55	55	51	54	56
University education (%)	20	22	41	20	13	29
Employed (%)	86	72	83	85	74	77
Using contraceptives (%)	78	74	84	–	–	69
Uncertain fertility intentions (%)	23	11	22	–	41	30
Social pressure (mean)	0.11	0.02	− 0.14	− 0.17	–	0.15
Expected costs (mean)	− 0.06	0.11	− 0.09	− 0.10	–	0.09
Expected benefits (mean)	− 0.01	0.02	− 0.02	0.02	–	− 0.02
N	2,492	3,616	1,956	3,290	3,530	3,047

Country	Intending a child within three years			Intending a child later than within three years			Not intending a child		
	DY	PY	Y	DY	PY	Y	DN	PN	N
b) Distribution of childbearing intentions in the sample of wave 1 and in the panel sample (in %), unweighted data									
*Wave* 1									
Austria	23	13		9	4		44	7	
Bulgaria	25	3		3	3		60	6	
France	19	15		4	5		54	3	
Hungary			18			18			54
Italy	10	20		7	4		42	17	
Poland	20	13		4	4		44	14	
*Panel sample*									
Austria	23	12		9	4		44	7	
Bulgaria	23	3		3	2		63	6	
France	20	14		3	5		55	3	
Hungary			18			17			55
Italy	10	20		7	4		42	17	
Poland	19	11		3	4		49	15	

^a^Respondents aged 21–45, fecund and with fecund partners, and with valid information on childbearing intentions. The panel sample is not exactly our analytical samples as, for the sake of a maximal consistency with the wave 1 sample, it does not exclude respondents who 1) changed partners or became single between the survey waves and 2) who did not have a child by wave 2

^b^The abbreviations should be read as follows: *DY* Definitely yes, *PY* Probably yes, *Y* Yes, *DN* Definitely not, *PN* Probably not, *N* No. Respondents aged 21–45, fecund and with fecund partners, and with valid information on childbearing intentions. The panel sample is not exactly our analytical samples as, for the sake of a maximal consistency with the wave 1 sample, it does not exclude respondents who 1) changed partners or became single between the survey waves and 2) who did not have a child by wave 2. Sample sizes are the same as in Table 4a

**Table 5:** Confirmatory factor analysis

Item (survey question)	Factor 1: Social pressure	Factor 2: Expected costs	Factors 3: Expected benefits
	Estimate (standard error)		
a) Parameter estimates			
Most of your friends think that you should have a/another child	1		
Your parents think that you should have a/another child	1.127 (0.007)		
Most of your relatives think that you should have a/another child	1.137 (0.006)		
Now, suppose that during the next 3 years you were to retire. I would like you to tell me what effect you think this would have on various aspects of your life:			
The possibility to do what you want		1	
Your employment		0.964 (0.015)	
Your financial situation		0.928 (0.014)	
What people around you think of you			1
The closeness between you and your partner			1.528 (0.022)
Certainty in your life			1.584 (0.024)
The closeness between you and your parents			1.243 (0.017)
The care and security they may get in old age			1.208 (0.019)
Covariances			
F1 ~ F2	− 0.194 (0.006)		
F1 ~ F3	0.199 (0.004)		
F2 ~ F3	− 0.097 (0.003)		

Degrees of freedom	Comparative Fit Index (CFI)	Tucker-Lewis Index (TLI)	Root Mean Square Error of Approximation (RMSEA)	RMSEA 90% confidence interval
b) Model fit statistics				
41	0.980	0.973	0.47	0.45–0.49

^a^An acceptable model has values of CFI and TLI above 0.9, and RMSEA below 0.5. The calculation of RMSEA includes a division by the degrees of freedom. Thus, if there are zero degrees of freedom, RMSEA is not calculated

^b^Possible answers to questions about social pressure on having a child: were: 1—strongly disagree, 2—disagree, 3—neither agree nor disagree, 4—agree, 5—strongly agree. Possible answers to questions about expected costs of having a child were: 1—much better, 2—better, 3—neither better nor worse, 4—worse, 5—much worse. Possible answers to questions about expected benefits of having a child were: 1—much worse, 2—worse, 3—neither better nor worse, 4—better, 5—much better

**Table 6:** Characteristics of *intended, sooner-than-intended* and *unintended* parents in East and West, weighted data for all six countries

	Intended		sooner-than-intended		unintended	
	West	East	West	East	West	East
Women (%)	53	54	48	55	46	54
Age (mean)	30.3	29.3	28.8	27.9	34.0	31.9
*Parity distribution*						
Childless (%)	50	45	51	28	11	1
With 1 child (%)	38	49	34	44	32	41
With 2 + children (%)	12	6	14	28	57	58
University education (%)	32	37	21	22	19	14
Employed (%)	84	83	87	68	75	73
Using contraceptives (%)*	63	58	79	78	78	66
Uncertain fertility intentions (%)**	38	14	50	63	53	36
Social pressure (mean)***	0.75	1.02	0.57	0.52	0.05	− 0.09
Expected costs (mean)***	− 0.41	− 0.28	− 0.21	− 0.06	− 0.07	0.12
Expected benefits (mean)***	0.15	0.26	0.14	0.11	− 0.03	− 0.15

Social pressure as well as the expected costs and benefits are standardised variables with mean at 0 and standard deviation equal to 1. Interpretation: as for Table 3. *N* = 2,067

*Italy and Hungary excluded as they did not asked about contraceptive use. *N* = 1,257

**Hungary excluded as the possible answers to all questions about fertility intentions were only *yes* and *no*. *N* = 1,665

***Italy excluded as it asked about social pressure in a way not comparable with other countries. *N* = 1,659

**Table 7:** Estimated ß-coefficients and their *p* values of becoming a* sooner-than-intended* or an *unintended* parent rather than an *intended* parent (ref.), binary logistic models

Ref.: Intended parents	M1		M2		M3		M4		M5		M6	
	beta	*p* val	beta	*p* val	beta	*p *val	beta	*p* val	beta	*p* val	beta	*p* val
*Sooner-than-intended*												
East (ref.: West)	0.62	0.09	− 0.22	0.69	0.04	0.95	0.10	0.86	0.27	0.65	− 0.11	0.85
Men (ref: Women)	1.60	0.12	1.88	0.08	2.08	0.06	2.26	0.04	2.15	0.07	2.46	0.04
Age	− 0.25	0.06	− 0.26	0.05	− 0.23	0.09	− 0.22	0.12	− 0.26	0.08	− 0.24	0.11
Age^2^	0.01	0.19	0.01	0.18	0.01	0.24	0.01	0.27	0.01	0.20	0.01	0.26
University education (ref.: secondary and below)	− 0.60	0.12	− 0.61	0.12	− 0.50	0.21	− 0.52	0.19	− 0.54	0.18	− 0.66	0.11
Being employed (ref: not working)	0.20	0.63	0.40	0.38	0.35	0.45	0.37	0.41	0.22	0.64	0.26	0.58
Using contraceptives	1.03	0.01	1.18	0.01	0.74	0.11	0.75	0.11	0.71	0.13	0.60	0.22
Parity (ref.: childless M1-M2, childless West M3-M6)												
1 child	− 0.02	0.97	− 0.37	0.51	− 0.38	0.51	− 0.36	0.53	− 0.57	0.33	− 0.50	0.39
2 + children	1.33	0.01	0.42	0.52	0.29	0.65	0.23	0.73	− 0.03	0.96	0.17	0.81
East × 1 child			0.95	0.24	0.71	0.39	0.65	0.43	0.62	0.46	0.64	0.45
East × 2 + children			2.61	0.01	2.15	0.03	2.12	0.03	2.10	0.03	2.18	0.03
Uncertain fertility intentions (ref: certain intentions)					1.17	0.00	1.12	0.00	1.04	0.01	0.93	0.01
Social pressure							− 0.14	0.48	− 0.01	0.95	0.08	0.72
Expected benefits									− 2.05	0.02	− 1.28	0.14
Expected costs											1.28	0.01
Men x East	− 0.63	0.22	− 0.60	0.51	− 0.69	0.45	− 0.75	0.42	− 1.01	0.28	− 1.03	0.29
Men x Age	− 0.02	0.93	0.01	0.98	− 0.05	0.81	− 0.06	0.77	− 0.05	0.80	− 0.04	0.84
Men x Age2	− 0.01	0.52	− 0.01	0.45	− 0.01	0.60	− 0.01	0.64	− 0.01	0.62	− 0.01	0.62
Men x University education	0.38	0.51	0.36	0.54	0.26	0.66	0.26	0.66	0.28	0.64	0.38	0.53
Men x Being employed	− 0.91	0.14	− 1.11	0.09	− 1.15	0.08	− 1.20	0.07	− 1.06	0.12	− 1.10	0.11
Men x Using contraceptives	0.21	0.74	− 0.04	0.95	0.24	0.73	0.21	0.76	0.16	0.82	0.12	0.87
Men × 1 child	0.65	0.26	0.76	0.33	0.74	0.35	0.62	0.44	0.75	0.36	0.89	0.28
Men × 2 + children	0.68	0.36	0.82	0.39	0.83	0.39	0.75	0.44	0.85	0.39	0.99	0.33
Men x East × 1 child			− 0.16	0.89	0.05	0.97	0.23	0.85	0.34	0.78	0.04	0.97
Men x East × 2 + children			− 0.40	0.78	− 0.06	0.97	0.01	0.99	0.15	0.92	0.09	0.95
Men x Uncertain fertility intentions					− 0.24	0.66	− 0.21	0.69	− 0.11	0.83	− 0.04	0.94
Men x Social pressure							− 0.10	0.72	− 0.15	0.61	− 0.24	0.44
Men x Expected benefits									0.97	0.43	1.40	0.29
Men x Expected costs											0.95	0.25
Intercept	− 2.82	0.00	− 2.77	0.00	− 3.06	0.00	− 3.02	0.00	− 2.54	0.00	− 2.32	0.00
Null deviance	581.35	581.35	581.35	581.35	581.35	581.35						
Residual deviance	501.96	489.92	473.72	471.94	464.31	444.79						
Generalised R2 (McFadden's)	0.14	0.16	0.19	0.19	0.20	0.23						
N	1,086											
*Unintended*												
East (ref.: West)	2.12	0.00	0.13	0.92	0.19	0.88	0.80	0.52	1.15	0.36	0.64	0.62
Men (ref: Women)	2.32	0.06	1.81	0.17	1.55	0.24	1.69	0.21	2.07	0.15	1.90	0.20
Age	− 0.09	0.47	− 0.11	0.40	− 0.10	0.44	− 0.06	0.67	− 0.06	0.69	0.03	0.86
Age^2^	0.01	0.12	0.01	0.09	0.01	0.11	0.01	0.16	0.01	0.17	0.01	0.42
University education (ref.: secondary and below)	− 0.88	0.01	− 0.88	0.01	− 0.88	0.01	− 1.10	0.00	− 1.06	0.01	− 1.09	0.01
Being employed (ref: not working)	0.40	0.25	0.51	0.17	0.51	0.17	0.68	0.08	0.52	0.21	0.38	0.40
Using contraceptives	0.74	0.02	0.81	0.01	0.77	0.02	0.73	0.03	0.62	0.08	0.47	0.22
Parity (ref.: childless M1-M2, childless West M3-M6)												
1 child	1.91	0.00	1.26	0.13	1.26	0.13	1.51	0.08	1.19	0.17	1.42	0.10
2 + children	4.30	0.00	2.93	0.00	2.93	0.00	2.64	0.00	2.08	0.01	2.35	0.00
East × 1 child			1.43	0.28	1.39	0.29	0.84	0.53	0.62	0.65	0.41	0.77
East × 2 + children			2.67	0.04	2.63	0.05	2.00	0.13	1.72	0.21	1.51	0.27
Uncertain fertility intentions (ref: certain intentions)					0.23	0.47	− 0.04	0.90	− 0.09	0.80	− 0.23	0.53
Social pressure							− 0.97	0.00	− 0.71	0.00	− 0.63	0.00
Expected benefits									− 3.74	0.00	− 2.51	0.00
Expected costs											2.15	0.00
Men x East	− 1.27	0.01	− 0.53	0.76	− 0.23	0.89	− 0.75	0.67	− 1.70	0.35	− 1.46	0.43
Men x Age	− 0.02	0.92	− 0.02	0.90	− 0.05	0.79	− 0.09	0.64	− 0.16	0.46	− 0.16	0.47
Men x Age^2^	0.00	0.74	0.00	0.74	0.00	0.91	0.00	0.99	0.00	0.70	0.00	0.69
Men x University education	0.29	0.57	0.41	0.43	0.23	0.66	0.37	0.50	0.52	0.37	0.56	0.35
Men x Being employed	− 1.14	0.03	− 1.26	0.02	− 1.56	0.01	− 1.62	0.01	− 2.09	0.00	− 1.76	0.02
Men x Using contraceptives	0.24	0.60	0.23	0.62	0.15	0.76	0.14	0.79	0.32	0.56	0.40	0.48
Men × 1 child	− 0.09	0.92	0.51	0.64	0.54	0.62	0.10	0.93	0.17	0.88	− 0.01	0.99
Men × 2 + children	− 0.75	0.39	− 0.32	0.76	− 0.43	0.69	− 0.47	0.67	− 0.32	0.78	− 0.43	0.71
Men x East × 1 child			− 0.78	0.67	− 0.96	0.60	− 0.22	0.91	0.49	0.80	0.73	0.71
Men x East × 2 + children			− 0.37	0.84	− 0.40	0.83	0.10	0.96	0.68	0.73	0.86	0.67
Men x Uncertain fertility intentions					1.25	0.01	1.48	0.00	1.67	0.00	1.85	0.00
Men x Social pressure							0.43	0.10	0.57	0.05	0.53	0.08
Men x Expected benefits									− 2.58	0.07	− 2.84	0.05
Men x Expected costs											− 0.47	0.54
Intercept	− 6.19	0.00	− 5.17	0.00	− 5.27	0.00	− 5.13	0.00	− 4.76	0.00	− 4.67	0.00
Null deviance	971.11	971.11	971.11	971.11	971.11	971.11						
Residual deviance	643.59	629.40	608.02	568.61	498.59	466.20						
Generalised R2 (McFadden's)	0.34	0.35	0.37	0.41	0.49	0.52						
N	1,174											

.

## Footnotes

1 Research has shown that pregnancy intentions include, in addition to rational considerations, emotional and psychological factors that are difficult to capture through survey questions. Thus, pregnancy intendedness should be perceived more as a continuum than as a simple dichotomy (Bachrach & Newcomer 1999); or as a concept with at least two dimensions, namely an affective dimension and a planning dimension (Stanford et al., 2000). However, as this study does not focus on analysing women’s perceptions of their pregnancy intendedness, we do not provide an in-depth discussion on this topic, and instead refer the reader to the respective literature (e.g. Arteaga et al., 2017; Santelli et al., 2003).

2 *Unintended* or *sooner-than-intended* (in italics) is equivalent to “classified as unintended or sooner-than-intended”. The italics are used to denote that we do not make assumptions about whether such births were unplanned at the time of conception; or whether they occurred following a change in intentions not captured by the panel survey, and were, in fact, intended. Analogically, *intended* is equivalent to “classified as intended”.

3 We restrict our analysis to these six countries because they applied comparable filters to questions about fertility intentions and used similar question framing. Other countries with GGS data available for two waves used different filters and/or noncomparable questions and precodes; see Beaujouan (2014) for details.

4 In Bulgaria, the data collection within the second wave started, in fact, two and a half years after the first wave.

5 In order to provide full evidence on the prevalence of *sooner-than-intended* and *unintended* births, in the Appendix we show descriptive analyses which include respondents who were single at wave 1 but partnered at wave 2 or changed partners between the waves.

6 We do not distinguish between married, cohabiting and LAT unions because there were not enough cohabiting and LAT couples in the samples in the post-socialist countries.

7 The remaining criteria, i.e. having the same partner at both survey waves and a child born between waves 1 and 2, are impossible to control for as they require information retrieved from wave 2.

8 The questions were not posed to respondents expecting a child at wave 1. In Italy, however, respondents were not asked about their own or their partner’s current pregnancy. Therefore, we excluded from the analysis those Italian respondents who must have known about their own or their partner’s pregnancy at the time of the interview; i.e. those who had a child within 26 weeks after the interview.

9 In France, the questions were asked in a different order and a slightly different form; for details please consult Brzozowska and Beaujouan (2020).

10 For a small number of respondents (around 1%), information on their short-term fertility intentions was missing (DK or NA), but information on their long-term fertility intentions was available. In such cases, we recoded short-term fertility intentions as negative and classified the respondents according to their long-term fertility intentions.

11 We also performed analyses for an observation window of 2.5 years (i.e. 30 months) for all countries, excluding all pregnancies reported at wave 2. The results did not differ from those presented here. Therefore, we have opted for the longer observation window, which yields more cases to analyse (i.e. more births between the waves).

12 The reason for why we did not use multinomial logistic regression is that, in the statistical software R, we could not get plausible average marginal effects for multinomial models. However, we compared the coefficients obtained from binary logistic models presented in this paper with those obtained from multinomial logistic models. There were no substantial differences (results available upon request).

13 In Appendix, Fig. 7 shows the numbers for the whole sample, irrespective of partnership status.

14 Figure 8 in Appendix compares these numbers with those for respondents who found a (new) partner between the survey waves. As expected, the numbers for the latter are much higher: the share of *sooner-than-intended* births more than doubles in the East and quadruples in the West as compared to respondents with the same partner at both waves. In contrast, the figures for *unintended births* do not follow this pattern: the numbers for women with the same and a new partner did not differ substantially, whereas for men they were slightly higher among those who reported a new partner at wave 2 as compared to those who had the same partner.

15 The difference between M6 and M5 is -6 p.p. with standard error equal 3.5 p.p. and is statistically significant at α = 0.05 (for computation method, see Gelman & Stern, 2006).
